# Immunogenicity of Novel Live Vaccine Based on an Artificial rHN20 Strain against Emerging Fowl Adenovirus 4

**DOI:** 10.3390/v13112153

**Published:** 2021-10-26

**Authors:** Yu Zhang, Qing Pan, Rongrong Guo, Aijing Liu, Zhuangzhuang Xu, Yulong Gao, Hongyu Cui, Changjun Liu, Xiaole Qi, Yanping Zhang, Kai Li, Li Gao, Xiaomei Wang

**Affiliations:** 1 State Key Laboratory of Veterinary Biotechnology, Harbin Veterinary Research Institute, Chinese Academy of Agricultural Sciences, Harbin 150069, China; zhangyu_vm@163.com (Y.Z.); panqing20050101@126.com (Q.P.); brook_grr@163.com (R.G.); liuaijing@caas.cn (A.L.); xuzhuangzhuang21@163.com (Z.X.); gaoyulong@caas.cn (Y.G.); cuihongyu@caas.cn (H.C.); liuchangjun@caas.cn (C.L.); qixiaole@caas.cn (X.Q.); zhangyanping03@caas.cn (Y.Z.); likai@caas.cn (K.L.); gaoli@caas.cn (L.G.); 2 Jiangsu Co-Innovation Center for the Prevention and Control of Important Animal Infectious Disease and Zoonoses, Yangzhou University, Yangzhou 225009, China

**Keywords:** fowl adenovirus 4, hepatitis-hydropericardium syndrome, rHN20, live vaccine, immunogenicity evaluation

## Abstract

In recent years, hepatitis-hydropericardium syndrome (HHS), caused by novel fowl adenovirus 4 (FAdV-4), has caused serious economic losses to the poultry industry. Vaccines are important for preventing and controlling HHS. Current FAdV-4 vaccine research and development are mainly focuses on inactivated vaccines and relatively fewer live vaccines. We previously demonstrated that the hexon gene is the key gene responsible for the high pathogenicity of FAdV-4 and constructed a non-pathogenic chimeric virus rHN20 strain based on the emerging FAdV-4. In this study, the immunogenicity of artificially rescued rHN20 was evaluated in chickens using different routes and doses as a live vaccine. The live rHN20 vaccine induced high titers of neutralizing antibodies against FAdV-4 and fully protected the immunized chickens against a lethal dose of FAdV-4. Furthermore, immunized chickens showed no clinical symptoms or histopathological changes in the FAdV-4-targeted liver, and the viral load in the tissues of immunized chickens was significantly lower than that of chickens in the challenge control group. Collectively, the live rHN20 vaccine effectively protected our sample against FAdV-4 infection and can be considered a live vaccine candidate for preventing HHS in the poultry industry.

## 1. Introduction

Fowl adenoviruses (FAdVs) are members of the *Aviadenovirus* genus and Adenoviridae family and are classified into five species (FAdV-A–E) and 12 serotypes (FAdV-1–7, 8a, 8b, and 9–11) [1]. The pathogenicity of different serotypes or even different strains of FAdVs is not completely consistent. Among the identified strains, FAdV-4 shows the highest virulence [2,3,4]. In 2015, severe hepatitis-hydropericardium syndrome (HHS) was observed in 3–5-week-old chickens and was determined to be caused by a novel FAdV-4 on chicken farms in China, resulting in mortality rates of 30–100% [5,6,7]. Recent studies showed that 7–180-day-old chickens infected with FAdV-4 exhibited clinical symptoms and mortality [8], and FAdV-4 infection has been reported in various countries worldwide [2,9].

Recent studies of FAdV-4 vaccines focused on inactivated vaccines, subunit vaccines, and genetically engineered vaccines have been reported [10,11,12,13]. In contrast, live FAdV-4 vaccines have not been widely examined. Live vaccines are developed based on either low-pathogenic or non-pathogenic strains. To date, three naturally non-pathogenic strains of FAdV-4 have been isolated: the ON1 strain (GenBank No. GU188428) isolated from Canada, KR5 strain (GenBank No. HE608152) isolated from Japan, and B1-7 strain (GenBank No. KU342001) isolated from India. However, the protection efficiency of these strains against FAdV-4 is unclear. Live attenuated FAdV-4 have been produced by passaging virulent FAdV-4 in chicken embryos [14] or QT35 cells [15,16], but the protective effect of these attenuated vaccines on the emerging FAdV-4 is also unknown. Recent studies revealed that recombinant viruses generated from novel genotype FAdV-4 strain in which enhanced green fluorescent protein (EGFP) was fused to the N-terminus of fiber-2 gene or in which the N-terminus of fiber-2 was knocked out was not pathogenic to specific pathogen-free (SPF) chickens, but the titers of the recombinant viruses were significantly lower in vitro than those of wild-type FAdV-4 [17,18].

We previously isolated the FAdV-4 virulent strain HLJFAd15 (GenBank No. KU991797) from chickens with severe HHS in Heilongjiang Province, China. HLJFAd15 showed a mortality rate of 100% in SPF chickens, and sequencing revealed a 1966-base pair fragment that had been naturally deleted at the right end of the genome compared to strains isolated earlier in other countries. Thus, this strain was identified as a novel genotype FAdV-4 [19]. Subsequently, we replaced the hexon of the HLJFAd15 strain with that of a natural non-pathogenic ON1 strain to obtain a recombinant FAdV-4 strain rHN20 with viral titers similar to those of the wild-type virus but without pathogenicity [20]. Here, we evaluated the protective efficacy of rHN20 as a live vaccine candidate. The immunization of rHN20 with different routes and doses conferred full protection against FAdV-4 challenge in SPF chickens. Our data advanced the development of an efficient live FAdV-4 vaccine and vaccine vector.

## 2. Materials and Methods

### 2.1. Cells and Viruses

Chicken Leghorn male hepatocellular (LMH) cells were maintained in Dulbecco’s modified Eagle’s medium/nutrient mixture F-12 ham (DMEM/F12) medium (Sigma-Aldrich, St. Louis, MO, USA) supplemented with 10% fetal bovine serum (Sigma-Aldrich, St. Louis, MO, USA) and incubated at 37 °C in 5% CO_2_.

The highly pathogenic FAdV-4 strain HLJFAd15 (GenBank No. KU991797) was isolated in our previous study [19]. The artificial non-pathogenic strain rHN20 is a chimeric virus generated by replacing its hexon gene with that of ON1 (GenBank No. GU188428) using HLJFAd15 as the backbone [20]. The recombinant FAdV-4 rWT-EGFP strain expressing EGFP was derived by inserting an EGFP expression cassette at the 1966-bp site of the natural deletion in the HLJFAd15 genome. All viruses were amplified in LMH cells and stored at –80 °C. The HLJFAd15 and rHN20 viral titers were titrated with plaque-forming units (PFU) in monolayer LMH cells. The median tissue culture infective dose (TCID_50_) was used to calculate the viral titer of rWT-EGFP according to the expression of EGFP. The titers of HLJFAd15, rHN20 and rWT-EGFP were 1.36 × 10^7^ PFU/mL, 3.14 × 10^7^ PFU/mL, and 10^7.67^ TCID_50_/mL, respectively.

### 2.2. Animals and Ethics Statement

SPF white Leghorn chickens were purchased from the Experimental Animal Center of Harbin Veterinary Research Institute (HVRI), Chinese Academy of Agricultural Sciences (CAAS), and housed in negative pressure isolators with adequate water, food, and light. Animal studies were approved by the Committee on the Ethics of Animal Experiments of HVRI, CAAS (Ethical permission code HVRI-IACUC-2021-0426-5) (Approval #5 from 26 April 2021) and performed according to international standards of animal welfare.

### 2.3. Animal Experiments

**Figure 1:**
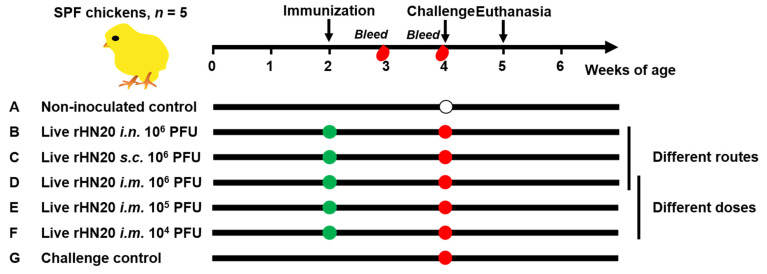
Schematic diagram of animal experiment design. SPF chickens were immunized at 2 weeks of age and blood was collected at 3 and 4 weeks of age. The chickens were challenged at 4 weeks of age and euthanized at 5 weeks of age. Seven groups with 5 chickens in each group were evaluated. Group (A) was neither immunized nor challenged as non-inoculated control group, and group (G) was not immunized but challenged as a control group. Groups (B–D) were immunized with 10^6^ PFU rHN20 through the intranasal (*i.n.*), subcutaneous (*s.c.*) or intramuscular (*i.m.*) routes. Groups (D–F) were immunized with different doses including *i.m.* 10^6^, 10^5^, and 10^4^ PFU. Among them, group (D) *i.m.* 10^6^ PFU was treated using different routes and doses.

As shown in Figure 1, 35 SPF chickens were randomly and equally divided into seven groups of five chickens each. Two groups of chickens were used as non-inoculated and challenged controls and were not immunized. Three groups of chickens were immunized with 10^6^ PFU rHN20 in 200 μL DMEM/F12 per bird via different immunization routes (intranasal, subcutaneous, and intramuscular) at 2 weeks of age. Two groups of chickens were intramuscularly immunized with different doses of rHN20 (10^5^ or 10^4^ PFU per bird) in 200 μL DMEM/F12 at 2 weeks of age. Blood samples were collected from all chickens at 3 and 4 weeks of age to separate the serum for determining the neutralizing activity against FAdV-4. All groups except for the non-inoculated controls at 4 weeks of age were challenged with 2000 PFU HLJFAd15 and observed for one week, during which daily illnesses and deaths were counted. Chickens from the non-inoculated control were administered an equivalent volume of DMEM/F12 in the same manner. The animal experiment was ended when the chickens reached 5 weeks of age, surviving chickens were euthanized, and organ tissues were collected for viral load detection and histopathological examination. Tissues from dead chickens in the challenge group were used as positive controls.

### 2.4. Virus-Neutralization Assay

The rWT-EGFP strain of FAdV-4 was used to detect serum neutralization activity against FAdV-4. Chicken serum samples were inactivated at 56 °C for 30 min, filtered through 0.22 µm filters, diluted at serial 2-fold ratios with DMEM/F12, and aliquoted into 96-well-plates at 100 μL per well. rWT-EGFP was diluted to 200 TCID_50_/mL in DMEM/F12 containing 2% fetal bovine serum, and 100 μL was added to each well of a 96-well-plate containing chicken serum, mixed, and incubated at 42 °C for 1 h. A monolayer of LMH cells in each well of a 96-well plate was seeded with 100 μL of the mixture and cultured in a constant temperature incubator at 37 °C for 6 days, after which EGFP was detected using an inverted fluorescence microscope (EVOS FL, Carlsbad, CA, USA).

### 2.5. Real-Time PCR to Determine the Tissue Viral Load

Total DNA was extracted from the liver, spleen, and kidney using an AxyPrep Body Fluid Viral DNA/RNA Miniprep Kit (Axygen, Union City, CA, USA) according to the manufacturer’s instructions. The fragment in the L1 region of the hexon gene of FAdV-4 was used as the target gene, and the chicken ovotransferrin gene was used as a reference gene. Real-time PCR amplification in a 20 μL reaction mixture was performed with Premix Ex Taq (probe qPCR) (TaKaRa, Shiga, Japan) and a QuantStudio 5 system (Applied Biosystems, Foster City, CA, USA). As described above, the final viral load concentration was presented as the copy number per 10^6^ cells [20,21].

### 2.6. Histopathological Examination

The liver from each group of chickens was immediately fixed in 10% formalin when the birds died or were euthanized. After 48 h, the fixed liver samples were embedded in paraffin wax, sectioned into 4–5 μm slices, stained with hematoxylin and eosin, and examined under an optical microscope (Leica DM4000B, Wetzlar, Germany).

### 2.7. Statistical Analyses

Data are reported as the standard errors of the mean (SEM) determined using two-way analysis of variance. Statistical significance was set at *p* < 0.05, and the results with different levels of significance are indicated with different asterisk numbers.

## 3. Results

### 3.1. Serum Neutralizing Activity of Vaccinated SPF Chickens against FAdV-4

**Figure 2:**
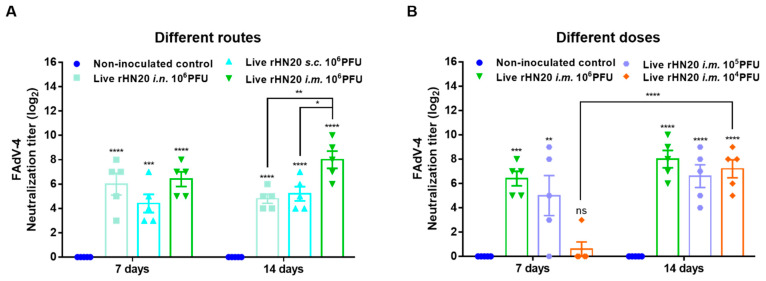
Neutralizing activity of vaccinated chicken serum towards FAdV-4. The neutralizing activity of immune serum after administration via different routes (**A**) and different doses (**B**) in chickens was detected using FAdV-4 strain rWT-EGFP in LMH cells at 7 and 14 days post-immunization. The data for the non-inoculated control group and *i.m.*10^6^ PFU group are shown in (**A**,**B**) are shared. Error bars represent the SEM. The significance of differences was determined using two-way analysis of variance, with *, *p* < 0.05, **, *p* < 0.01, ***, *p* < 0.001, ****, *p* < 0.0001, and ns, not significant. Routes for immunization were: *i.n.* = intranasal, *s.c.*= subcutaneous, and *i.m.* = intramuscular.

Two-week-old SPF chickens were immunized via different routes and with different doses of the rHN20 live vaccine candidate, and the neutralizing activity of the serum against FAdV-4 was examined at 7 and 14 days after immunization (Figure 1). After immunization of the chickens by different routes with 10^6^ PFU rHN20, the chicken serum showed robust neutralizing activity against FAdV-4 at 7 and 14 days. The neutralizing activity of the intramuscular injection group was significantly higher than that of the intranasal and subcutaneous injection groups at 14 days post-vaccination (Figure 2A). Although neutralizing activity was detected in the chicken serum at different immunization doses at 14 days post-vaccination, only the 10^6^ and 10^5^ PFU groups showed neutralizing activity at 7 days post-vaccination, whereas only one bird from group the 10^4^ PFU group exhibited neutralizing activity (Figure 2B).

### 3.2. Protective Efficacy against Lethal FAdV-4 Challenge in SPF Chickens

**Figure 3:**
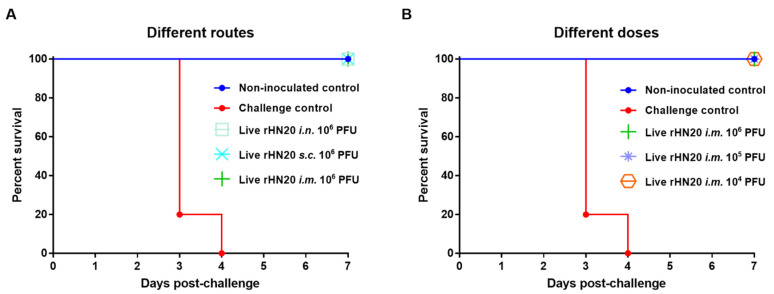
Protective efficacy of live rHN20 vaccine candidate against FAdV-4 challenge. Two weeks after immunization, all chickens were challenged with a lethal dose of FAdV-4. The survival rates of pre-immunized chickens via different routes (**A**) and different doses (**B**) were observed for 7 days. Data for the non-inoculated control group, *i.m.* 10^6^ PFU group, and challenge control are shown in (**A**,**B**) are shared. Routes for immunization were: *i.n.* = intranasal, *s.c.* = subcutaneous, and *i.m.* = intramuscular.

To evaluate the protective efficacy of the rHN20 live vaccine candidate, all groups of SPF chickens were challenged with 2000 PFU FAdV-4 via intramuscular injection at 14 days post-vaccination and observed for 7 days. None of chickens in all immunization groups and in the non-inoculated control group showed any clinical symptoms, such as feed intake. Whereas chickens in challenge control group showed rough feathers, and decreased feed intake, and all chickens died within 4 days after challenge. Thus, immunization via different routes (Figure 3A) or doses (Figure 3B) afforded 100% (5/5) protection of SPF chickens from lethal challenge with the virus.

### 3.3. Viral Load in Different Tissues of SPF Chickens

**Figure 4:**
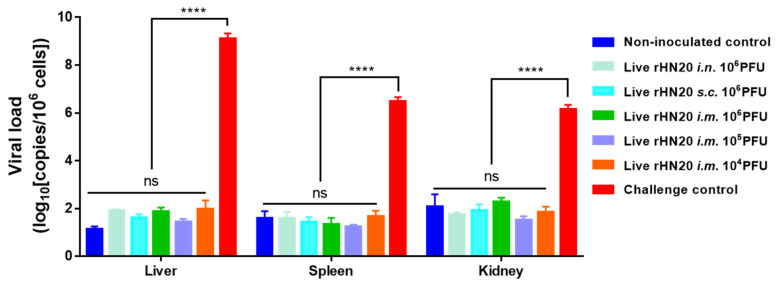
Viral load residues in tissues of chickens in different groups. Surviving chickens were euthanized at 7-days post-challenge for viral load detection using real-time PCR in the liver, spleen, and kidney. Tissues from challenge control group were used as positive controls. Three chickens per group were tested. Error bars represent the SEM. The significance of differences was determined using two-way analysis of variance (****, *p* < 0.0001, ns, not significant). Routes for immunization were: *i.n.* = intranasal, *s.c.* = subcutaneous, and *i.m.* = intramuscular.

At the end of the experiment, the viral load in the liver, spleen, and kidney tissues of surviving chickens was measured using real-time PCR (Figure 4). High copy numbers of FAdV-4 were detected only in the viscera of dead chickens in the unimmunized challenge control group, whereas the remaining groups showed background values comparable to those in the non-inoculated controls, regardless of the immunization route and dose number.

### 3.4. Histological Examination of Liver

**Figure 5:**
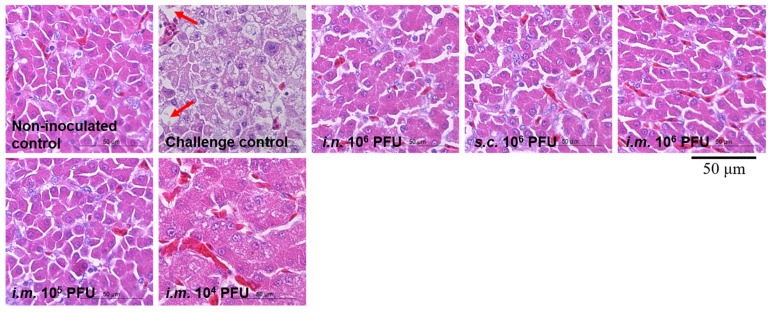
Histological examination of liver from chickens challenged with FAdV-4. Large necrotic focal areas and steatotic vacuoles were present in chicken liver cells from the challenge controls, whereas each immunization group was indistinguishable from the non-inoculated controls. Solid arrows indicate steatotic vacuoles. Scale bar, 50 μm. Routes for immunization were: *i.n.* = intranasal, *s.c.* = subcutaneous, and *i.m.* = intramuscular.

Severe hydropericardium and typical FAdV-4-related gross liver lesions were observed only in the challenge control chickens and not in immunized chickens. Liver samples from chickens in different groups were further examined by histopathological examination (Figure 5). Large necrotic focal areas and steatotic vacuoles were present in chicken liver cells from the challenge controls, whereas each immunization group was indistinguishable from the non-inoculated controls. These results suggest that live rHN20 not only prevents chicken death due to FAdV-4 infection but also protects against liver damage.

## 4. Discussion

Vaccines are effective for preventing and controlling viral diseases in humans and animals. Live vaccines exhibit similar characteristics as those of natural infection by the virus, allowing them to induce strong immunity to prevent disease. Examples of successful live attenuated vaccines include those for poliovirus [22], measles virus [23], and equine infectious anemia virus [24]. Live attenuated vaccines for SARS-CoV-2 [25] and African swine fever virus [26] are also being studied. Since the outbreak of HHS caused by novel FAdV-4, in 2015, several candidate vaccines have been reported [19]. However, few live attenuated vaccines based on the emerging novel FAdV-4 have been evaluated [17,18]. We previously generated a non-pathogenic chimeric virus rHN20 by replacing the hexon gene of the FAdV-4 virulent strain HLJFAd15 with that of the non-pathogenic strain ON1 [20]. In this study, the immunogenicity of artificially rescued rHN20 was evaluated in SPF chickens after administration via different routes and doses for use as a live vaccine. SPF chickens vaccinated with live rHN20 showed complete protection against FAdV-4 infection. The live vaccine based on rHN20 can not only provide sufficient protection to chickens, but also reduces vaccine costs because of the lack of antigen purification and adjuvant addition compared with inactivated vaccines.

Antigen-specific neutralizing antibodies in the serum are important indicators of the efficacy of most virus vaccines. The FAdV-4 inactivated vaccine, subunit protein vaccine, and recombinant virus-vectored subunit vaccine induce neutralization activity in the serum [18,19]. Here, all chickens immunized with rHN20 via different routes or with different doses produced robust neutralizing antibodies against FAdV-4. However, the cellular immunity of the rHN20 vaccine requires further investigation. Recently, a fiber-2 edited live vaccine against FAdV-4 was reported to provide full protection to chickens, but the artificial modification significantly reduced the viral titers of the rescued strain, largely limiting the clinical application of the candidate vaccine [18]. However, the non-pathogenic vaccine candidate rHN20 strain used in this study showed an equivalent viral titer as the wild-type FAdV-4, which is beneficial for the commercial production and large-scale promotion of vaccines.

FAdVs are double-stranded DNA viruses with a liner genome length of approximately 43–45 kb, highlighting its potential capacity to insert and deliver foreign genes, just like herpesviruses [27,28], poxviruses [29], and lentiviruses [30]. Mammalian adenoviruses have been developed into mature viral vectors, such as adenovirus-vectored Ebola and the recently emerging SARS-CoV-2 [31,32,33]. Among the FAdVs, FAdV-1 and FAdV-9 have been preliminarily explored as vaccine vectors, although limited progress in their development has been made [34,35]. To the best of our knowledge, FAdVs with serotypes other than serotype 4 do not protect chickens against HHS induced by pathogenic FAdV-4 infection. Therefore, studies aimed at developed an FAdV-4 vector are urgently needed. The live vaccine derived from chimeric rHN20 protected chickens against HHS and its high viral titers, at similar levels as the wild-type strain, providing an important foundation for the development of a novel FAdV-4-based vector. The FAdV-4 vectored vaccine (using rHN20 as the vector bone) prevented not only HHS induced by virulent FAdV-4, but also the disease corresponding to the presenting antigen.

## 5. Conclusions

In conclusion, the immunogenicity of the artificial hexon-replaced chimeric FAdV-4 strain rHN20 was evaluated for use as a live vaccine. This live vaccine induced high titers of neutralizing antibodies against FAdV-4 and completely protected chickens from FAdV-4 infection. The live vaccine developed in this study should be examined as a vaccine candidate against HHS in the poultry industry, and the rHN20 strain can be used to develop FAdV-4 vectored bivalent or multivalent vaccines.

## Data Availability

All data are included in the manuscript.
